# Effect of Zirconia Dental Implant Surfaces on Bone Integration: A Systematic Review and Meta-Analysis

**DOI:** 10.1155/2017/9246721

**Published:** 2017-02-16

**Authors:** Ali Hafezeqoran, Roodabeh Koodaryan

**Affiliations:** Department of Prosthodontics, Faculty of Dentistry, University of Medical Sciences, Tabriz, Iran

## Abstract

*Background*. The information available about osseointegration and the bone to implant interaction of zirconia implants with various surface modifications is still far from sufficient.* Objective*. The purpose of this systematic review and meta-analysis was to evaluate and compare zirconia dental implants with different surface topographies, with a focus on bone to implant contact and removal torque.* Methods*. The systematic review of the extracted publications was performed to compare the bone to implant contact (BIC) with removal torque (RT) values of titanium dental implants and machined and surfaced modified zirconia implants.* Results*. A total of fifteen articles on BIC and RT values were included in the quantitative analysis. No significant difference in the BIC values was observed between titanium and machined zirconia implants (*p* = 0.373; 95% CI: −0.166 to 0.443). However, a significantly better BIC values were observed for acid etched zirconia implants compared with those of titanium implants (*p* = 0.032; 95% CI: 0.068 to 1.461). Unmodified zirconia implants showed favorable BIC values compared to modified-surface zirconia implants (*p* = 0.021; 95% CI: −0.973 to −0.080).* Conclusion*. Acid etched zirconia implants may serve as a possible substitute for successful osseointegration.

## 1. Introduction

Commercially pure titanium and some of its alloys have so far been the material of choice in implant dentistry and orthopedics [1]. However, the gray color of titanium impairs esthetic results particularly in the presence of thin mucosal biotype [2]. In recent years, high strength zirconia implants have emerged as an alternative to titanium implants and provide better esthetic outcomes [3]. Yttrium-stabilized tetragonal zirconia is a well-studied bioinert structure which provides high strength, fracture toughness, esthetic, and biocompatibility [4].

Osseointegration is biological fixation of implant relating to direct bone to implant contact (BIC) without an intervening connective tissue layer [5]. BIC is regarded as key indicator for successful osseointegration which governs the overall success and survival of implants [6]. Moreover, it is clearly understood that the surface properties of a biomaterial play a fundamental role in osseointegration process [7]. Implant material composition and surface topography influence the wound healing processes following the implantation and subsequently affect osseointegration [8]. A moderately rough surface topography is known to positively affect the interfacial tissue reaction [9]. Therefore, numerous surface modification methods have been proposed to enhance osseointegration and improve the success rates. Such approaches mainly include optimization of the surface microroughness (sandblasting or acid etching), application of the bioactive coatings (calcium phosphate, bisphosphonate, and collagen), sintering particles onto the implant surface, nanotechnology, and laser technology [10–14]. Although there has been considerable discussion of zirconia surface modification and structure, the information available about osseointegration responses and the implant-bone interaction of these implants is still far from sufficient. Thus, the optimal surface topography for a dental implant remains unclear [15].

The present systematic review and meta-analysis was conducted to evaluate BIC around zirconia implants with different surfaces. The null hypotheses are as follows. (1) There are no differences in BIC for machined zirconia dental implants compared with titanium implants and (2) there are no differences among zirconia dental implants with different surface modifications with regard to BIC and RT.

## 2. Methods

### 2.1. Structure of the Review

This systematic review adheres to the criteria of the “Preferred Reporting Items for Systematic Review and Meta-Analysis” (PRISMA) [16].

### 2.2. Search Strategy

Electronic searches without time restrictions were performed in the PubMed database for relevant publications until 15 June 2016. The following search terms were used in this study: dental implant, zirconia implant, zirconia dental implant, zirconia osseointegration, removal torque values, histomorphometry, zirconia surface treatment, and dental implant surface treatment with OR and AND. Authors also manually searched the literature for relevant publications in* British Journal of Oral and Maxillofacial Surgery, Clinical Implant Dentistry and Related Research, Clinical Oral Implants Research, European Journal of Oral Implantology, Implant Dentistry, International Journal of Oral and Maxillofacial Implants, International Journal of Oral and Maxillofacial Surgery, International Journal of Periodontics and Restorative Dentistry, International Journal of Prosthodontics, Journal of Clinical Periodontology, Journal of Dental Research, Journal of Dentistry, Journal of Oral Implantology, Journal of Craniofacial Surgery, Journal of Cranio-Maxillofacial Surgery, Journal of Maxillofacial and Oral Surgery, Journal of Oral and Maxillofacial Surgery*, and* Journal of Periodontology.*

### 2.3. Eligibility Criteria

The studies were included if they met the following inclusion criteria: (1) animal studies, (2) publication in the international peer reviewed literature, (3) English language, and (4) histological (animals) assessment of BIC and RT.

Exclusion criteria were (1) animal studies with less than two animals per observation or group, (2) computational studies, (3) human studies, (4) studies that evaluated only one type of implant surface, and (5) reviews.

Based on population, intervention, control, and outcome (PICO) criteria, the focused questions were as follows. (1) Does the choice of implant material affect bone to implant contact when comparing titanium to zirconia in animal models? (2) What is the impact of zirconia implant surface modification on BIC and RT when comparing modified to as sintered zirconia in animal models? The study population was the animals which received machined or surface-modified zirconia and titanium dental implants. The comparison was made between titanium and machined zirconia, between titanium and surface-modified zirconia implants, and between machined and surface-modified zirconia implants. The two outcomes evaluated were the BIC and RT of implants.

### 2.4. Study Selection

The titles were screened independently by the two reviewers. The abstracts of all studies were assessed for relevance to the review and those appearing to meet the inclusion criteria were retrieved. Also, all reference lists of the selected studies and relevant reviews were checked manually to identify additional articles that have been missed in database searches. Disagreements were settled by discussion between the authors until a consensus was achieved.

### 2.5. Quality Assessment

All studies were assessed for quality depending on whether they met all the quality criteria or if one or more criteria were partially met or not met using the SYRCLE's risk of bias tool [17]. This tool contains 10 entries which facilitates bias judgment. “Yes” indicates a low risk of bias and “no” indicates a high risk of bias, while” unclear” means insufficient information to assess the risk of bias.

### 2.6. Summary Measures and Synthesis of Results

In the majority of included studies, the animals were subdivided into various groups. The comparison was performed for different implant materials and surface treatments. These data were recorded separately during the review to be incorporated into the analysis. Thus the population of the study was more than indicated by the numbers of the included studies.

The meta-analysis was based on the Mantel–Haenzel and Inverse Variance methods. Bone to implant contact and the removal torque were the continuous outcome measures which were expressed in standard difference in mean. A fixed model was used to calculate the weighted means at 95% confidence intervals (CI). The values were considered significant when *p* < 0.05. The data were analyzed using comprehensive meta-analysis software version 2 (Biostat Inc., Englewood, New Jersey, USA).

## 3. Results

### 3.1. Literature Search

**Figure 1:**
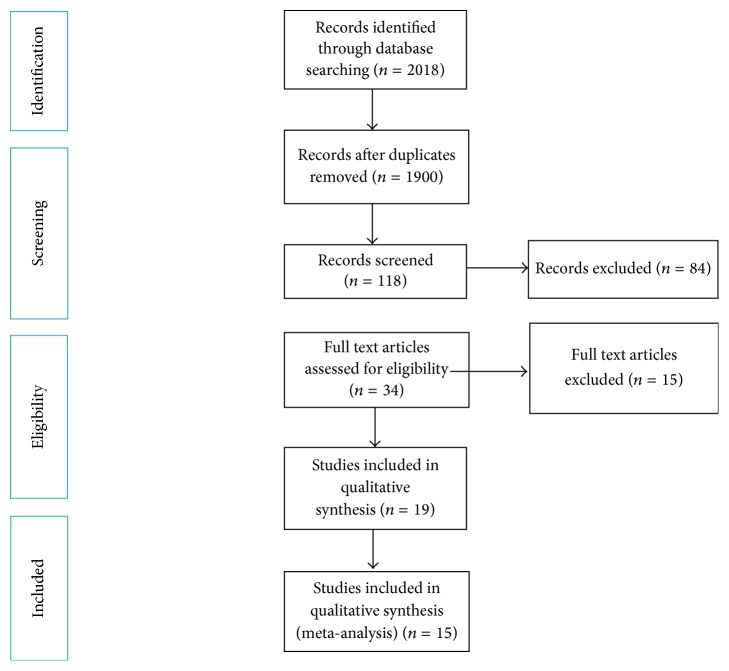
Diagram of the search strategy according to PRISMA statement.

The search in the database retrieved an initial number of 2018 references. 1900 titles were excluded after further assessment (Figure 1). All selected publications were subdivided according to differences in implant materials and surface treatments into 3 groups: (1) studies that assessed the impact of the machined or surface-modified zirconia implant on BIC in comparison to titanium implants, (2) studies that evaluated the BIC of powder injection mold (PIM) zirconia implants, and (3) studies that compared RT of machined zirconia with surface-modified zirconia implants (Table 1). After the application of the inclusion/exclusion criteria and qualitative assessment of these publications, 15 studies were considered for a quantitative meta-analysis (Tables 1 and 2).

### 3.2. Meta-Analysis

A total of 15 studies were included in this quantitative meta-analysis which were published from 2004 to 2015. Seven of the selected studies [14, 18–23] evaluated the BIC values of machined zirconia and titanium surfaces. Three studies compared BIC values of titanium and etched zirconia implants [6, 24, 25] and 3 other studies [14, 20, 26] assessed those of titanium and blasted zirconia implants. Machined and surface-modified zirconia implants were considered in 3 studies [14, 18, 21]. PIM-treated and untreated zirconia implants were installed in 4 studies [15, 27–29]. Five studies compared RT values of zirconia with modified zirconia implants [14, 15, 27–29].

#### 3.2.1. Bone to Implant Contact (BIC)

Fifteen studies assessed the mean BIC values (%) around the implants in different observation periods. The range of BIC values for titanium, machined zirconia, blasted zirconia, and surface etched zirconia implant groups in selected studies was 31.80% to 87.85%, 33.74% to 84.17%, 41.35% to 67.4%, and 51.1% to 71.4%, respectively.

**Figure 2:**
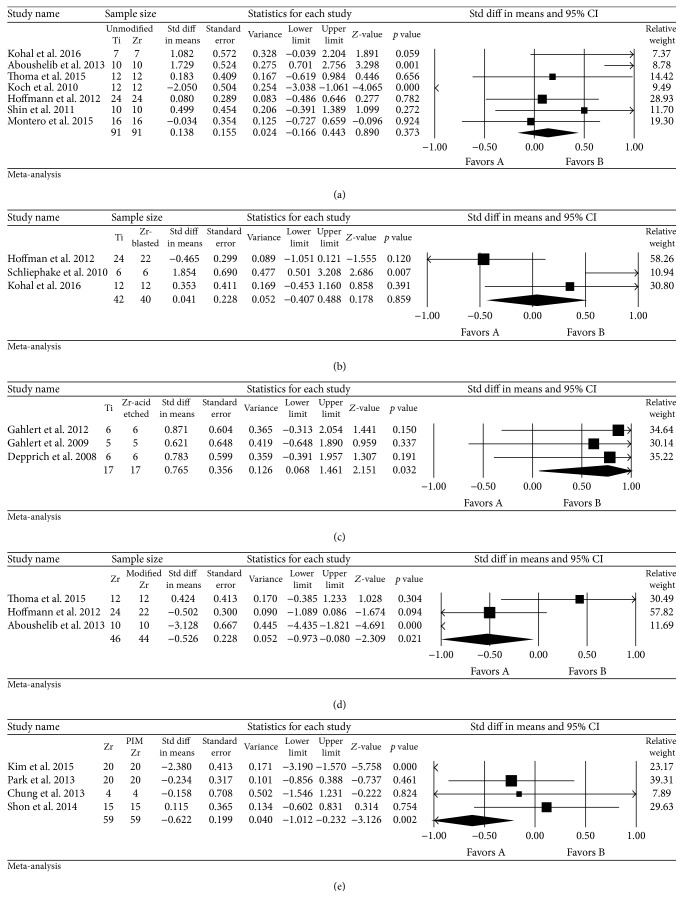
Forest plot for the event “BIC” in the comparison between titanium and zirconia implants (a), between titanium and sandblasted zirconia (b), between titanium and acid etched zirconia (c), between surface-modified and machined zirconia implants (d), and between untreated and PIM roughened mold zirconia implants (e).

The results of the studies suggested no significant difference in the BIC values between titanium and machined zirconia (standard difference in mean: 0.138, 95% CI: −0.166 to 0.443; *p* = 0.373) and between titanium and blasted zirconia implants (standard difference in mean: 0.041, 95% CI: −0.407 to 0.488; *p* = 0.859). However, a significant increase was observed in BIC values of acid etched zirconia compared with titanium implants (standard difference in mean: 0.766, 95% CI: 0.068 to 1.461; *p* = 0.032). Comparative studies of machined and surfaced modified zirconia implants showed a greater BIC for unmodified zirconia implants (standard difference in mean: −0.526, 95% CI: −0.973 to −0.080; *p* = 0.021). In addition, PIM untreated zirconia implants showed significantly greater BIC than PIM-treated zirconia implants (standard difference in mean: −0.622, 95% CI: −1.012 to −0.232; *p* = 0.002) (Figure 2).

#### 3.2.2. Removal Torque (RT)

**Figure 3:**
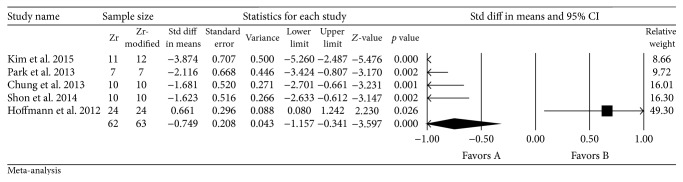
Forest plot for the event “RT” in the comparison between surface-modified and unmodified zirconia implants.

All five accepted studies showed more favorable RT values for untreated zirconia implants than those of machined zirconia implants, which were statistically significant (standard difference in mean: −0.749, 95% CI: −1.157 to −0.341; *p* < 0.001) (Figure 3).

## 4. Discussion

This systematic review and meta-analysis focused on 2 questions. (1) Does the choice of the implant material affect BIC when comparing titanium to zirconia in animal models? (2) What is the impact of zirconia implant surface modification on BIC and RT when comparing modified to as sintered zirconia in animal models? Data synthesis showed a significantly better BIC values for acid etched and unmodified zirconia implants compared with those of titanium and modified-surface zirconia implants respectively. Thus, the null hypotheses were rejected.

It is well known that surface topography, chemistry, and roughness affect the rate and quality of new tissue formation [30]. Surface modification can enhance bone healing and integration of titanium implants and result in higher bone-implant contact ratios [20, 31]; however, the interfacial interaction of modified zirconia with bone is still not fully understood. A number of animal studies have been published that outline hard and soft tissue integration of zirconia implants on the histological level and have shown promising results [29, 32, 33]. Further, cell culture studies have demonstrated that zirconia especially with a moderately rough surface is accepted by osteoblasts and integrates into bone tissue [9, 34]. Scarano et al. [35] examined the bone-implant interface of machined zirconia implants at 4 weeks of healing and reported BIC values of 68.4%. Similarly, Akagawa et al. [36] observed a BIC ratio of 66% to 81% for zirconia implants inserted into mandible of monkeys after 24 months of healing which was similar to that of zirconia implants at the 12-month observation. It is noteworthy to mention that the current review included only those studies comparing unmodified or modified zirconia with titanium implants in order to perform a direct comparison. The present meta-analysis found an equivalent BIC values for machined zirconia compared with titanium implants. The lack of significant difference between the zirconia and titanium implants could lead to the conclusion that the zirconia implant is as osteoconductive as the titanium implant. For titanium implants, roughened surfaces have demonstrated superiority compared to their smooth, machined predecessors [7, 9]. Consequently, zirconia implants with a roughened surface have been suggested to be capable of achieving greater stability in bone than machined zirconia implants [29]. Gahlert et al. confirmed that the increased surface roughness of sandblasted and acid etched zirconia implants not only has an important influence on bone integration but also is associated with increased removal torque strength and bone stability in minipigs [6]. Zirconia implants with rough surfaces have also demonstrated higher removal torque resistance in rabbits [18]. The osseointegration capacity of machined zirconia surface is substantially increased after modification by sandblasting [20]. However, Gahlert et al. [6] specified that further improvements in the surface roughness of zirconia implants are needed. As the review only included animal studies, the analysis of each surface modification was hindered by the limited number of studies, and only the two surface modifications (acid etching or blasting) were compared with titanium implants. According to the meta-analysis, no significant difference was found in the BIC values between titanium and blasted zirconia implants; however, the surface modification for the acid etched zirconia surfaces resulted in significantly higher BIC compared with the titanium implants. The evaluated histological data were commonly in agreement with this result in which the modified zirconia implants were comparable to titanium implants. However, Gahlert et al. [6] and Depprich et al. [24] demonstrated that the BIC values obtained for the entire implant thread length had no significant difference between modified zirconia and titanium implants in minipig models during the observation periods. Thus, the authors concluded that there is an evidence for a better outcome of surface-modified zirconia implants over titanium implants.

When comparing the BIC values of modified zirconia surfaces relative to the machined ones, data synthesis has identified that unmodified zirconia surface may be favored over modified zirconia implant. This result was supported by the RT values which also favored to zirconia implants. When evaluating the outcomes reported in these studies, it must be emphasized that mean BIC values assessed after respective healing periods were commonly in agreement with this result. Accordingly, the bone to implant contact around zirconia implants exhibited greater values than those of the modified zirconia implants [14, 21]. Conversely, Aboushelib et al. [18] reported greater bone to implant contact for selective infiltration etched zirconia implants after healing periods (65.38 ± 5.7% at 4 weeks and 75.01 ± 5.1% at 6 weeks), confirming a better bone reaction to modified zirconia. However, it is not considered correct to present comparative data without defining surface roughness. Even a machined zirconia surface may vary considerably in roughness as is the case for blasted, acid etched, or other modified surfaces. Thus different results may be reported of the same surface topography [37, 38]. Unfortunately, detailed information on surface topography of all included studies was not given. This makes it increasingly difficult to compare different study outcomes particularly when the techniques used for surface modifications vary considerably. Also, the manufacturing technique and the chemical and physical composition of zirconia implants show substantial differences [39]. Hence, a surface that is termed rough in one study may be termed smooth in another. In addition, tissue response to altered surface topography need not necessarily reflect the performed change of the surface alone. When the surface topography is changed, the surface chemistry or physics may change simultaneously [40]. These factors seem to play important roles in the osseointegration of modified zirconia implants, although it is not yet clear which are most important. So although tendencies for improved osseointegration following zirconia implant modification were seen, further researches and experiments should focus on these materials.

It can be concluded that an acceptable BIC is achieved after the healing period independent of implant material and surface treatment. There is a general consensus that roughening the implant surface above the level seen with most machined surfaces enhances the bone response to implants. However, the effects of surface roughness on the cell function and interplay between a zirconia implant and the adjoining bone tissue have to be clearly investigated.

Some of the challenges in the translation of animal studies are decreased by meta-analysis of animal studies; however, the results have to be interpreted with caution because of the presence of several confounding factors. The available literature on bone-implant interface involves a wide range of studies with various methods and results; thus the impact of one surface over another can barely be compared [41]. Arguably, the most insidious source of confounding is the method of bone to implant contact assessment. By far, histomorphometric analysis has been the gold standard in evaluating the bone-implant interface. The various methods of the histomorphometric analysis, microscopic magnification, and different software and algorithms appear to play a crucial role in BIC calculations which may subsequently lead to deviant results [29, 32]. The bone tissue has to be transferred to specialized laboratory for preparation immediately after animal sacrifice. Besides the various methods of fixation used in research protocols, preparation of thin tissue and fixation of animal tissue in formalin may disturb the results. Moreover, variation in the area to be evaluated or region of interest exists among the studies. Obviously, the values obtained from the entire implant surface may characterize the performance in the clinically realistic context [25]. However, different regions of interest like the whole implant surface, the mineralized zone between two threads, and the contact at the best three threads were reported in evaluated studies.

Another important issue to consider is the animals used for the experiment. Pigs and rabbit were commonly used as experimental models in accepted studies, while monkey and rat were used in two studies. Variation in animal models among studies may result in considerable variations in the results [32]. Selection of an appropriate animal model for demonstrating the response of bone tissue to biomaterial is difficult, mainly because the bone characteristics, microstructure, composition, modeling, and remodeling are different from those of humans [40]. While dogs, sheep, goats, pigs, or rabbits are suitable models for evaluating implants (according to international standards), no species fulfils the requirements of an ideal model. It is important to consider the research question when selecting the animal model. The characteristics of human bone are best approximated by dog models; but substantial differences exist in the bone anatomy, microstructure, and remodeling between rabbit and humane [42]. Rat is unsuitable due to significant dissimilarities in bone structure [43].

Considering these limitations, the findings of the current study should be interpreted cautiously. Several other confounding factors such as surface topography, chemistry, roughness, implant design and dimension, and healing time influence the results. Coexistence of multiple factors in the studies makes the evaluation of one particular factor impossible and the lack of control over these factors lowers the potential of definitive result extraction.

## 5. Conclusion

Within the limitation of the present study, the following conclusions can be drawn.

(1) No significant difference in the BIC values was observed between titanium and zirconia and between titanium and sandblasted zirconia implants, (2) a significantly better BIC was observed for surface treated zirconia compared with titanium implants, (3) unmodified zirconia implants showed favorable BIC values compared to machined zirconia implants; moreover, PIM untreated zirconia implants showed significantly better results than PIM-treated zirconia implants, and (4) untreated zirconia implants showed favorable RT values compared to machined zirconia implants.

## Figures and Tables

**Table 1:** Characteristics of studies included in review.

Study, year	Animal	Implant no, dimensions (mm^2^ × mm)	Manufacturer	Material	Surface treatment	BIC (%)	RT (N/cm)
(Thoma et al. 2015)	Dog	48				6 months:	
		4 × 8	VITA Zahnfabrik	One piece Zr	—	87.71 ± 25.07	—
		3.7 × 9	Metoxit	One piece Zr	Microporous	78.58 ± 17.26	—
		4.1 × 8	BPI	Two piece Zr	Nanostructured and hydrophilic	84.17 ± 25.07	—
		3.3 × 8	Straumann	One piece Ti	Sandblasted and etched	87.85 ± 13.59	—

(Kim et al. 2015)	Rabbit					4 weeks:	
		64	Dentime	Zr	Machined	32.15 ± 10.76	19.44
		4 × 7	Cetatech	Zr	PIM roughened	58.38 ± 11.28	57.63

(Park et al. 2013)	Rabbit					4 weeks:	
		80	Chaorum	Ti	Machined	42.54 ± 10.26	10.56 ± 6.03
		4 × 7	Cetatech	Zr	PIM untreated	61.63 ± 12.39	44.24 ± 8.41
			Cetatech	Zr	PIM roughened	64.42 ± 11.45	64 ± 35 ± 10.46

(Chung et al. 2013)	Rabbit	100				4 weeks:	
		4 × 7	Cetatech	Zr	PIM untreated	59.59 ± 11.50	45 ± 63 ± 10.78
			Cetatech	Zr	PIM roughened	61.52 ± 12.96	64.99 ± 12.21

(Shon et al. 2014)	Rabbit					4 weeks:	
		100	Cetatech	Zr	PIM untreated	58.26 ± 10.09	39.7 ± 11.69
		4 × 7	Cetatech	Zr	PIM roughened	56.93 ± 12.95	59.21 ± 12.35
			Cetatech	Zr	PIM untreated + He plasma	70.87 ± 9.11	46.75 ± 13.15
			Cetatech	Zr	PIM roughened + He plasma	72.27 ± 10.31	60.98 ± 12.70

(Gahlert et al. 2012)	Minipig	36	Straumann AG	Zr	Hydrofluoric acid	4 weeks: 70 ± 14.5	—
		4.1 × 10				8 weeks: 67.1 ± 21.1	—
						12 weeks: 68.3 ± 22.8	—
			Straumann AG	Ti	SLA	4 weeks: 64.7 ± 9.4	—
						8 weeks: 68.3 ± 22.8	—
						12 weeks 83.7 ± 10.3	—

(Montero et al. 2015)	Dog	32				5 months	
		3.8 × 8	Metoxit AG	Zr	Machined	57 ± 15.2	—
			Microdent	Ti	—	56.5 ± 14.4	—

(Hoffmann et al. 2012)	Rabbit	96	*Z*-system AG	Zr	Machined	6 weeks: 32.996 ± 14.192	35.409 ± 9.063
		3.25 × 6				12 weeks: 33.746 ± 14.529	40.591 ± 17.081
			*Z*-system AG	Zr	Laser modified	6 weeks: 39.965 ± 13.194	26.309 ± 11.415
						12 weeks: 43.87 ± 14.544	39.708 ± 9.819
			*Z*-system AG	Zr	Sandblasted	6 weeks: 39.614 ± 15.029	19.590 ± 12.128
						12 weeks: 41.350 ± 15.816	28.727 ± 18.766
			—	Ti	Acid etched	6 weeks: 34.155 ± 15.816	39.818 ± 14.093
						12 weeks: 34.818 ± 12.209	51.909 ± 16.149

(Aboushelib et al. 2013)	Rabbit	60	—	Zr	Machined	4 weeks: 53.30 ± 4.2	—
		3.7 × 8				6 weeks: 62.14 ± 2.8	—
			Zimmer Dental	Ti	SLA	4 weeks: 56.93 ± 3.9	—
						6 weeks: 68.31 ± 4.2	—
			—	Zr	Selective infiltration	4 weeks: 65.38 ± 5.7	—
					etching	6 weeks: 75.01 ± 5.1	—

(Koch et al. 2010)	Dog	48				4 months:	
			—	Zr	Machined	59.11 ± 7.45	—
			—	Zr	TiO2 coated	55.83 ± 13.92	—
			—	Ti	Sandblasted	40.91 ± 10.11	—
			—	Peek	—	26 ± 8.9	—

(Shin et al. 2011)	Rabbit	20				6 weeks:	
		3.5 × 6.6	—	Zr	Machined	26 ± 17.2	18.2 ± 2.69
			—	Ti	Machined	35.8 ± 21.8	10.9 ± 7.82

(Gahlert et al. 2009)	Pigs	30	Straumann AG	Zr	Hydrofluoric acid	4 weeks: 27.1 ± 3.5	—
		4.1 × 10				8 weeks: 51.9 ± 14	—
						12 weeks: 51.1 ± 12.4	—
			Straumann AG	Ti	SLA	4 weeks: 23.5 ± 7.5	—
						8 weeks: 53.3 ± 27.6	—
						12 weeks: 58.5 ± 11.4	—

(Schliephake et al. 2010)	Minipig	72	SPI EIEMENT	Zr	Sandblasted	4 weeks: 57.5 ± 14.3	4 weeks: 55.9 ± 18.4
		4.2 × 8				13 weeks: 54.6 ± 17.6	13 weeks: 99.4 ± 30.9
			—	Zr	Sandblasted and etched	4 weeks: 66.7 ± 15.8	4 weeks: 111.8 ± 42.4
						13 weeks: 57.6 ± 23.7	13 weeks: 100.3 ± 47
			—	Ti	Sandblasted and etched	4 weeks: 69.3 ± 17	4 weeks: 244.5 ± 34.9
						13 weeks: 78.9 ± 5.8	13 weeks: 221.9 ± 27.1

(Depprich et al. 2008)	Minipig	48	—	Zr	Acid etched	1 week; 35.3 ± 10.8	—
		3.5 × 9				4 weeks: 45.3 ± 15.7	—
						12 weeks: 71.4 ± 17.8	—
			—	Ti	Acid etched	1 week; 47.7 ± 9.1	—
						4 weeks: 58.6 ± 9.5	—
						12 weeks: 82.9 ± 10.7	—

Kohal et al. 2016	Monkey	24				9 months:	
		4 × 13	—	Ti	SLA	72.9 ± 14	—
		4 × 15	—	Zr	Sandblasted	67.4 ± 17	—

**Table 2:** Results of quality assessment.

Quality questions	studies														
	Montero et al. 2015	Thoma et al. 2015	Kim et al. 2015	Park et al. 2013	Chung et al. 2013	Shon et al. 2014	Ghahlert et al. 2012	Hoffmann et al. 2012	Aboushelib et al. 2013	Koch et al. 2010	Shin et al. 2011	Gahlert et al. 2009	Schliephake et al. 2010	Depprich et al. 2008	Kohal et al. 2016
Was the allocation sequence already generated and applied?	Yes	Yes	Unclear	Yes	Yes	Yes	Yes	Yes	yes	Unclear	Yes	Yes	Unclear	Unclear	Yes

Were the groups similar at baseline or were they adjusted for confounders in the analysis?	Yes	Yes	Yes	Yes	Yes	Yes	Yes	Yes	Yes	Yes	Yes	Yes	Yes	Yes	Yes

Was the allocation to the different groups adequately concealed during the experiment?	Unclear	Unclear	Unclear	Unclear	Unclear	Unclear	Unclear	Unclear	Unclear	Unclear	Unclear	Unclear	Unclear	Unclear	Yes

Were the animals randomly housed during the experiment?	Unclear	Unclear	Unclear	Yes	Unclear	Unclear	Unclear	Unclear	Unclear	Unclear	Unclear	Unclear	Unclear	Unclear	Unclear

Were the caregivers and/or investigators blinded from knowledge which intervention each animal received during the experiments?	Unclear	Yes	Unclear	Unclear	Unclear	Unclear	Unclear	Unclear	Yes	Unclear	Unclear	Unclear	Unclear	Unclear	Unclear

Were animals selected at random for outcome assessment?	Unclear	Yes	Unclear	Yes	Unclear	Yes	Unclear	Unclear	Unclear	Unclear	Unclear	Unclear	Unclear	Unclear	Unclear

Was the outcome assessor blinded?	Unclear	Unclear	Unclear	Unclear	Unclear	Unclear	Unclear	Unclear	Yes	Unclear	Unclear	Unclear	Unclear	Unclear	Yes

Were incomplete outcome data adequately addressed?	Yes	Yes	Yes	Yes	Yes	Yes	Yes	Yes	Yes	Yes	Yes	Yes	Yes	Yes	Yes

Are reports of the study free of selective outcome reporting?	Yes	Yes	Yes	Yes	Yes	Yes	Yes	Yes	Yes	Yes	Yes	Yes	Yes	Yes	Yes

Was the study apparently free of other problems that could result in high risk of bias?	Yes	Yes	Yes	Yes	Yes	Yes	Yes	Yes	Yes	Yes	Yes	Yes	Yes	Yes	No
